# A framework for managing health research capacity strengthening consortia: addressing tensions and enhancing capacity outcomes

**DOI:** 10.1136/bmjgh-2022-009472

**Published:** 2022-10-03

**Authors:** Nadia Tagoe, Justin Pulford, Sam Kinyanjui, Sassy Molyneux

**Affiliations:** 1 Office of Grants and Research, Kwame Nkrumah University of Science and Technology, Kumasi, Ghana; 2 Department of Global and International Health, Kwame Nkrumah University of Science and Technology, Kumasi, Ghana; 3 Department of International Public Health, Liverpool School of Tropical Medicine, Liverpool, UK; 4 Training, KEMRI-Wellcome Trust Research Programme, Kilifi, Kenya; 5 Nuffield Department of Medicine, Oxford University, Oxford, Oxfordshire, UK; 6 Health Systems and Research Ethics, KEMRI-Wellcome Trust Research Programme, Kilifi, Kenya

**Keywords:** Health policy, Health systems, Qualitative study

## Abstract

There has been a steady increase in health research capacity strengthening (HRCS) consortia and programmes. However, their structures and management practices and the effect on the capacity strengthening outcomes have been underexamined. We conducted a case study involving three HRCS consortia where we critically examined the consortia’s decision-making processes, strategies for resolving management tensions and the potential implications for consortia outcomes. We conducted 44 in-depth interviews with a range of consortia members and employed the framework method to analyse the data. We assessed the extent to which consortia’s management practices and strategies enabled or hindered research capacity strengthening using a capacity development lens. At the heart of consortium management is how tensions are navigated and the resolution strategies adopted. This study demonstrates that the management strategies adopted by consortia have capacity strengthening consequences. When deciding on tension management strategies, trade-offs often occur, sometimes to the detriment of capacity strengthening aims. When management strategies align with capacity development principles, consortium management processes become capacity strengthening mechanisms for participating individuals and institutions. Such alignment enhances programme effectiveness and value for money. Drawing on these findings, we propose an evidence-informed management framework that consortia leaders can use in practice to support decision-making to optimise research capacity gains. Considering the increasing investment in HRCS consortia, leveraging all consortium processes towards capacity strengthening will maximise the returns on investments made.

**What is already known on the topic**

- There has been a steady increase in health research capacity strengthening consortia and programmes, but their structures and management practices have been underexamined.

**What this study adds**

- Identifying, unpacking and managing tensions are crucial components of consortium management.
- Management decisions in research capacity strengthening consortia are complex and have capacity development consequences.
- Consortium management is a capacity strengthening mechanism in its own right.

**How this study might affect research, practice or policy**

- Consortium management processes and practices impact consortia’s capacity outcomes and must be adequately planned for, resourced and tracked.

## Introduction

Low and middle-income countries (LMICs) bear a significant proportion of the global disease burden.^1 2^ The COVID-19 pandemic has re-emphasised the critical role of health research capacity in countries' self-sufficiency, preparedness and ability to address both endemic and emerging health challenges.^3–5^

Several calls have been made in recent decades to prioritise health research capacity strengthening (HRCS) through national health research systems, increased partnerships and financing.^6–9^ Although this has contributed to a steady increase in HRCS consortia and programmes, there is little evidence on their effectiveness.^10 11^ To ensure optimised impact and sustained relevance, it is essential to continuously evaluate and improve these initiatives. Thus, assessing consortia’s outputs and outcomes needs to be coupled with understanding the processes and factors driving the outputs and outcomes.^12 13^ This is particularly crucial for complex interventions like HRCS programmes, which often involve multiple actors and components operating at multiple levels over extended periods.^13 14^ The performance and capacity outcomes of consortia are dependent on several factors. For instance, organisational capacity requires a combination of ‘hardware’ (such as infrastructure, staff, technology and finances); ‘tangible software’ (such as management knowledge and skills and organisational systems and procedures) and ‘intangible software’ capacities (such as communication, values and norms, relationships and power).^15–17^

Research capacity strengthening consortia have primarily focused on enhancing the hardware. Although some efforts have been made to address issues such as power and equity, both tangible and intangible software elements have received little attention in HRCS consortia.^18^ The effects of HRCS consortia management practices on their capacity strengthening goals have, therefore, been underexamined.^18^ This is a critical gap; consortium management involves complex processes of coordinating partners, activities and institutional systems, with potentially significant implications for programme outcomes. Moreover, management capacity is vital to the sustenance of science systems.^19^ Directors of capacity building consortia are often established scientists who are not necessarily trained managers,^20^ and management of capacity-strengthening consortia differs from the management of organisations or even research consortia. Furthermore, there is an increase in LMIC-led consortia to address power asymmetries and promote local research agenda setting.^11 21 22^ Thus, examining consortium management practices and the capacity strengthening impact in these contexts is vital.

In a precursor study, we identified and examined the following management processes of 10 LMIC-led HRCS consortia: selecting partners, determining consortia goals, assigning roles and responsibilities, instituting governance structures and processes, managing partners, allocating resources and coordinating activities.^20^ In so doing, we discovered that navigating tensions between divergent management strategies is central to consortium management.^20^

In this paper, we examine management tensions in consortia in more depth, including how tensions are addressed and how strategies adopted during consortium management processes enable or hinder capacity development. We then draw on the findings to propose a conceptual framework to guide management decision-making in capacity strengthening consortia.

## Methods

### Study design and setting

Following the precursor study,^20^ we conducted a more in-depth qualitative case study of three HRCS consortia. The consortia were part of Developing Excellence in Training Science and Leadership (DELTAS) Africa phase 1 (2016–2021), an Africa-led initiative aimed at strengthening health research capacity through enhancing scientific quality, research training, scientific citizenship and research management and environment.^23^ This initiative was of particular interest as it comprised 11 LMIC-led programmes, 10 of which were consortia.

**Figure 1:**
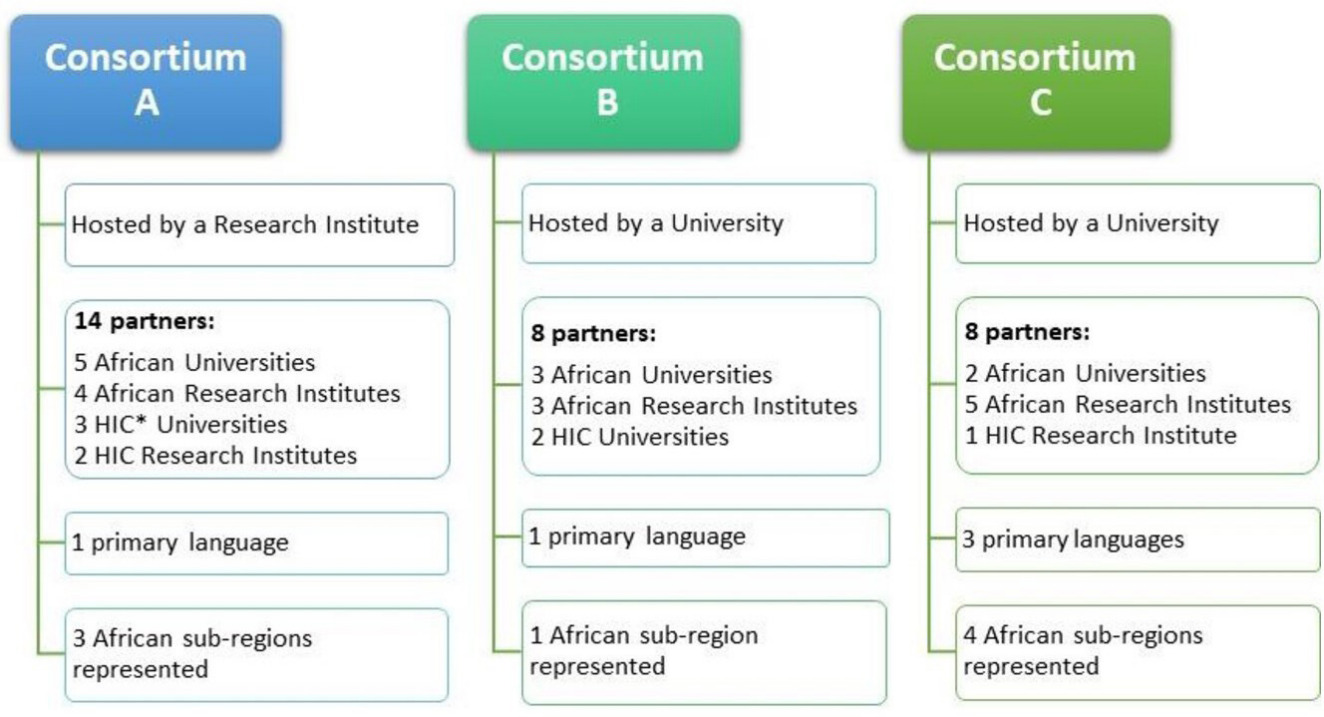
Characteristics of the three consortia cases. HIC, high-income country.

A stepwise approach was used to select consortia, institutional and individual participants for the study. First, three consortia were purposively selected as cases (figure 1). Elements of theoretical sampling^24^ and maximum variation sampling^25^ were employed to enable the examination of concepts that emerged from the precursor study in more depth and to capture diverse perspectives and contexts. Selection criteria included: (1) consortium characteristics such as size, subject focus and geographical and language diversity; (2) management approaches used such as centralised or decentralised management and (3) type of lead institution such as university or research institute. Second, the lead and three partner institutions with different levels of research capacity were selected from each consortium to capture multiple perspectives. Third, individuals from these lead and partner institutions who held key managerial roles in the consortium were selected as participants.

### Data collection and analysis

Data were collected by the first author (NT) through face-to-face in-depth interviews conducted with 44 participants (18, 14 and 12, respectively, from the three cases), including consortium directors, programme managers, partner lead representatives, finance officers and monitoring and evaluation officers (table 1). Participants included almost all stakeholders who played key management roles in the selected consortia. All participants approached agreed to participate and gave written informed consent. A semistructured in-depth interview guide was used (see online supplemental material) with interviews lasting for 60 min to 100 min and held in an office or meeting room. The interviews focused on exploring if and how consortia’s capacity strengthening aims influenced their management decisions and the tensions encountered in the process. All interviews were audio-recorded, and consortium and participant identifiers replaced with descriptor codes during transcription and data cleaning. Interview summaries were developed from the transcripts and notes taken by NT during the interviews. Interviews continued until thematic saturation was reached, whereby the same set of issues were being raised by participants (although with different examples and presentations) and no new themes were emerging.

10.1136/bmjgh-2022-009472.supp1 Supplementary data

**Table 1:** Participant distribution across cases

Type of participant	Case A	Case B	Case C	Total
Consortia directors	1	1	1	3
Partner lead representatives	3	2	4	9
Programme managers	2	2	1	5
Finance officers	2	4	4	10
M&E officers	1	3	1	5
Other consortium and institutional staff	8	1	1	10
HIC partner leads	1	1	*	2
**Total**	**18**	**14**	**12**	**44**

*One of the LMIC partner leads was also affiliated to and represented the HIC partner institution.

HIC, high-income country; LMIC, Low and middle income country.

The interviewer (NT) was introduced to the consortia directors by the DELTAS funders and had engaged the consortia in the precursor study.^20^ We recognised that this had the potential for the study to be perceived as a consortium evaluation exercise by participants, with implications for power dynamics and data quality. We therefore, took an explicit colearning stance throughout the research process and constantly reassured participants that the study was independent of the funders and not intended to evaluate their performance. NT also had several years of experience in managing HRCS consortia, which could have been a source of personal and professional biases and assumptions. The study team, including experienced biomedical and social science researchers with extensive experience in the African context, therefore acted as ‘peer debriefers’,^26^ supporting continuous reflexivity on the potential influence of positionality. Our approach to ensuring equitable participation in all study phases and publication authorship is outlined in our author reflexivity statement (see online supplemental material)

10.1136/bmjgh-2022-009472.supp2 Supplementary data

We used NVivo V.11 software to manage the data and facilitate data analysis, which was led by the first author (NT) and supported by all authors. We used framework analysis due to its systematic nature and appropriateness for case comparison and policy-oriented and practice-oriented research.^27 28^ Both deductive and inductive approaches were used in developing a thematic framework made up of themes and subthemes and based on both a priori themes from the research questions (in part based on the precursor study^20^ and the theoretical underpinning outlined below) and themes emerging from the data. We then applied the thematic framework to each transcript using NVivo and created a chart for each category of identified themes which were further clustered into cases. This step enabled more interpretive abstraction of concepts, meanings and patterns from the data and helped establish connections within and between identified themes and cases and between the data and existing literature and practice. Preliminary findings were shared with consortia leaders for verification.

### Theoretical underpinning

The essence of HRCS consortia is capacity development. Thus, we applied a capacity development lens in analysing the consortium management practices and strategies to determine their impact on research capacity strengthening (RCS).

This lens views research capacity as a systemic phenomenon that relies on the complex interaction between many factors and levels.^29–31^ Research capacity comprises multiple dimensions such as skills, leadership, strategy, infrastructure, management systems, collaborations and culture^32 33^ at individual, institutional and environmental levels.^34–36^ This concept is inconsistent with the widely used ‘planned approach’ to RCS, which employs result-based management methods such as logical framework analysis and focuses on output accountability. The planned approach reduces the complex capacity development process into linear cause-and-effect relationships between inputs, outputs and outcomes.^37 38^ Yet, sustainable capacity development is characterised by internally driven changes arising from multiple interactions among relevant actors within local systems, local ownership and experimentation and learning.^17 39–41^

## Results

### Tensions encountered and resolution strategies adopted by consortia

All three consortia reported similar tensions, yet differing resolution strategies were adopted. We discuss four main inter-related tensions encountered by consortia and the respective resolution strategies (table 2).

**Table 2:** Tensions encountered by consortia and strategies adopted

Tension	Illustrative quote	Consortia strategies		
		Consortium A	Consortium B	Consortium C
1. Individual or collective interests	‘You are dealing with different people, different backgrounds with different resources and you always have this fine line to find between the interest of the group and each one’s interest… If you are not careful, you will break the group’ (consortium C, lead institution, R1). ‘A very dramatic example… We said, “what level of training should we focus on for researchers, postdoc, PhD, Masters, interns?” And of course, different people seated around the table, representatives of different institutions, had their views… One of those would even bang the table to say, "we are not interested in postdocs first of all, because we don't have PhDs… that’s not our priority. If our institution is to move forward, we want Masters training”, with a bang on the table. And you can see that was an interest driven by the peculiarities of the institution’ (consortium B, lead institution, R2).	Common goal Individual training of researchers	Two-level goals Collective+partner-specific Individual+institutional	Tailored goals Partners select goals from a range based on need Individual+infrastructure
*2*. Efficient programme delivery or effective capacity strengthening	‘Should we go for second-tier, third-tier, or first-tier universities? Should we go for universities that have a lot of funding and resources or should we go for universities which have nothing? We spent a lot of time in identifying our partners’ (consortium A, lead institution, R2). ‘It is a tricky situation because you want to present a proposal that is competitive against others and so you are debating… if you want a very competitive application, take the best institutions. Of course, everything else remaining equal, those are likely to be winners… But when you start bringing in other considerations, you want to bring the weaker ones; you want to all move together; then the situation becomes a little tricky’ (consortium B, lead institution, R2).	Selection of partners with higher levels of capacity Focus on one research capacity component—training individual researchers Centralised partner management system	Selection of partners with varying levels of capacity Focus on multiple research capacity levels and components—individual, institutional, technical, managerial Decentralised partner management system	Selection of partners with varying levels of capacity Focus on multiple research capacity levels and components—individual, institutional, technical, managerial Decentralised partner management system
3. Excellence or equity	‘The DELTAS always talk about excellence, and even at the onset, they wanted to start with institutions that were excellent. So, if we were to form the consortium in the spirit of DELTAS, then we probably would have a smaller consortium where we would just bring those who are already high up there. In our situation, we didn't want to leave people behind because they were not excellent’ (consortium C, lead institution, R1). ‘It’s clear that if you put too much in the weaker institutions, it’s not going to be absorbed easily. So, in the governance discussions… should we allocate equal opportunities finance-wise… should we say equal number of PhDs for different institutions? And arguments can go either way. The weaker institution will say we have a greater need therefore we should have more PhDs… it’s a valid argument’’ (consortium B, lead institution, R2).	Merit-based fellow selection with a cap on the number of awards per partner	Merit-based fellow selection	Merit-based fellow selection with regional and gender balancing
4. Shared power or greater control	‘They [Directors] are quite influential in terms of making decisions… There are sometimes a bit of, what can I say, executive decisions being made. But again, you know when you think of any organization, if it’s completely 100% democratic, decisions are made very slowly, and sometimes there is not a lot of accountability. So, you need a bit of executive decision-making where the buck stops, and I’ve seen that happen in the management board’ (consortium A, partner institution, R5). ‘Everything relies on the PI [Director)… So, he still has some room to manoeuvre, which for me is good, it’s not bad. We cannot have more than one person being ‘responsible’ for stuff; then things will never get done’ (consortium C, partner Institution, R4).	Two-tier governance: Steering board and annual general meeting Centralised management	All-inclusive steering board Decentralised management	All-inclusive steering board Decentralised management

#### Tension 1: addressing individual or collective interests

Consortia leaders were confronted with diverse interests of individual partners and the collective consortium. Each partner had specific priorities based on their perceived capacity needs. For example, when discussing the programme goals, some partners advanced the need for PhD training, others for masters and others for infrastructural capacity. However, budgetary and time limitations meant that not all goals could be pursued. Decisions were mainly influenced by consortia’s interpretation of RCS and how different capacities were valued.

Each consortium adopted a different approach in addressing this tension (table 2). One consortium chose to focus on only individual researcher training for all partners (which we term ‘common focus’). Another adopted what we call the ‘two-level goals’ approach where consortia had collective goals and partners were also allowed to set locally relevant goals. The third consortium allowed partners to set ‘tailored goals’ based on their unique circumstances.

If institutions are going to be very interested in being part of the consortium, they must see benefits that relate to their own institutions (consortium B, lead institution, R2).

The consortium goals have been built to take into account the breadth of those needs… some have just MScs, because that’s where their needs are, and others have MScs and PhDs, and others have MScs, PhDs and postdocs, and others have a bit of all of that and also require sophisticated infrastructure… so we have been sort of providing for the whole array of needs (consortium C, lead institution, R1).

#### Tension 2: prioritising efficient programme delivery or effective capacity strengthening

Consortia often deliberated on the dilemma between efficient programme delivery and effective strengthening of partners’ capacity needs. This tension was experienced during several management processes, including selecting partners, determining consortia goals and activities, allocating resources and managing partners. For instance, when selecting partners, consortia leaders were torn between high-performing institutions or those with greater capacity gaps. Leaders reckoned that having ‘stronger’ partners would enhance the consortium’s efficiency, performance and competitiveness in subsequent funding applications. On the other hand, consortia were mindful of DELTAS capacity strengthening aim of building up less-capacitated partners on the continent.

Consortium A prioritised existing capacity in selecting partners to enable delivery of notable outputs within the grant period.

We are only dealing with first-tier universities… We were very deliberate about that because we don't want to start from 100 kilometres [meaning 'a long way back']. At least they were already running so let’s run with those (consortium A, lead institution, R2)

Consortia B and C aimed for balance by selecting both ‘stronger’ and ‘weaker’ partners to enable both programme performance in terms of measurable outputs and strengthening of less-capacitated partners.

The director said, “okay, this is the map of Africa… we need to be wide and to cover the different regions of Africa and also the different languages” …We started with people who were… already working on that topic, and then the second layer… But the third layer was more from countries that were not doing much research (consortium C, partner institution, R3).

Opting for a mix of institutions with varying strengths was not without its challenges and posed the risk of delivering suboptimal results. However, the leaders reasoned that it was worth taking that risk for the sake of strengthening partners’ capacity.

We wanted a mix of ‘stronger’ institutions and some which were not so strong in research… Without that consideration, we would have gone for just the strongest institutions… because we know they’ll deliver… But we took the risk and said, “let’s have the less-strong in order to build their capacity”. Our interest as a network was to pull everybody up as we move. So those were very important considerations (consortium B, lead institution, R2)

In determining programme goals, the consortia focused more on individual researcher training than institution-level capacity building, although to different degrees. For example, Consortium A leaders argued that, compared with institutional systems, similar levels of investment in individual fellowships produced more measurable outputs in shorter periods. Thus, in addressing the tension between efficient delivery of outputs and effective partner capacity strengthening, consortium A prioritised the former.

It’s easy to start with fellows… maybe it’s a low-hanging fruit. It’s easy; you can easily organize something and count. Data systems, for instance, are hard to count… For the same amount that you can use to train 500 fellows, maybe you can set up 10 data systems (consortium A, lead institution, R2).

Consortia B and C also prioritised individual researcher training but paid slightly more attention to strengthening institutional-level capacity. The leaders noted that effective capacity strengthening needed to be multilevel. Overall, the emphasis on individual capacity meant that potential benefits to less-capacitated partners such as institutional training capacity or research infrastructure were forfeited. This emphasis appeared to have been influenced by funder expectations and evaluation indicators that prioritised tangible outputs within the project period.

DELTAS has got those pillars of the theory of change… Of course, there’s a lot of emphasis on numbers; you know, publications, amount of funding, and so forth (consortium B, lead institution, R2).

The tension between efficient output delivery and effective capacity strengthening was also evident in partner management strategies. Consortium A chose a primarily centralised approach where the lead institution largely coordinated activities and financial transactions. Consortia B and C used a primarily decentralised system where partners managed their own work plans and budgets through subawards. Consortium A leaders noted that partner institutions had varying management capacities and gaps and attempting to tackle those gaps would adversely affect the consortium’s ability to deliver on its primary aim of training fellows.

We knew that the financial systems were really problematic, and we didn’t want to be dealing with financial issues as opposed to dealing with the primary functions of the consortium. So, we said, let’s first push and get out the fellows… that process alone is capacitating to the partner institutions… Otherwise, we would spend 50 percent of the time chasing money… because you know the bureaucracy of the Universities can be problematic… (consortium A, lead institution, R2).

Similar management gaps and the associated performance risk existed in consortia B and C institutions. Yet, their leaders asserted that decentralised management systems enabled the strengthening of both scientific and managerial capacity at individual and institutional levels and facilitated sharing of capacity benefits among all partners.

We decided to share the responsibility… and the resources… It’s also a way of improving the capacity in these places… they have to be involved not just as participants, but playing a more active role in running an aspect of the programme… So, having this decentralised system sort of spreads or… contributes to the overall lifting of the research environment in these places (consortium C, lead institution, R1)

In addition, there was multidirectional peer learning as lead institutions learnt from partners and partners learnt from each other.

It’s good because this gives us a new experience to manage money… we are discovering new procedures… that is the benefit of the management of co-applicants (consortium C, lead institution, M2).

Irrespective of the approach used, consortia leaders acknowledged that the tension between the options and the consequences of their choices were issues they had to continuously deal with.

It’s good to build that capacity within the institutions, so one can argue that. But I know after I’ve managed programmes where you subcontract to people, it’s usually a nightmare sometimes to report. So, there are pros and cons. (Consortium A, Partner Institution, R5)

On the one hand, because of the bureaucracy, it [decentralised approach] kind of delays how fast you want to do things, but on the other hand, sometimes that’s the price you’ll have to pay if you are going to build capacity. You’ll have to be patient with the systems (consortium B, lead institution, M2).

#### Tension 3: focusing on excellence or equity

Consortia encountered tensions between excellence and equity, particularly during partner selection and resource allocation processes. Consortia had to choose high-performing partners or aim for capacity equity, which required focusing on partners with the greatest needs. This tension was particularly highlighted in resource sharing and awarding of training fellowships. Leaders were torn between prioritising excellence through a merit-based system and equity through a quota-based system. All three consortia studied adopted the merit-based approach, which granted awards based on open competition within consortia. Some leaders argued that although ‘weaker’ partners had greater needs, they often could not deliver on outputs even when given the opportunity due to their capacity constraints, such as limited pools of potential trainees and supervisors. Although funder expectations and consortia competitiveness inclined leaders towards excellence-oriented decisions, consortia acknowledged that it was necessary to ensure some equity even within the excellence framework. Thus, consortia incorporated measures such as capping partner benefits, levelling out geographical and gender disparities and providing additional resources for other partner-specific needs (table 2).

If you want to keep the group together and make it sustainable, then bear in mind that everybody wants something out of it, and the whole group wants to move… You need to always… take that into your decision-making and in how you orient resources (consortium C, lead institution, R1).

#### Tension 4: prioritising shared power or greater control

Another source of tension was the power balance across partners. Shared power within the consortia meant promoting inclusive decision-making and good collaborative practice, while greater control meant greater influence by lead institutions and quicker decision-making processes. While acknowledging the essence of shared power in consortia, the pressure of accountability heightened leaders’ need to have greater control over decisions. Consortia’s perception of shared power depended on whether it was being considered in tangible or intangible terms. For instance, a tangible demonstration of shared power was the equal representation of partners on management boards. In one consortium where partner representation was limited to avoid having a large board, the partners raised concerns about having a voice and ownership.

We have a way of balancing that power by having the partners being members of the governing board… so, they, in reality, are the ones that set the pace of what the lead does… and if we don’t do it well, they feedback to us through the board (consortium C, lead institution, R1)

Another tangible demonstration of greater control and shared power was the use of centralised and decentralised partner management systems. Participants using the centralised system reported greater control of operations by the lead institution. With this approach, consortia bypassed systemic challenges in partner institutions, encountered fewer grant reporting challenges and averted performance risks. However, it resulted in missed opportunities for strengthening partners’ capacity. Partners also felt detached from management functions, engendering a diminished sense of ownership of consortia goals and limited institutional embeddedness.

The disadvantage of the centralised system is that the partners do not really grow. They are dependent on the capacity of the lead institution… The capacity is still at minimal levels… [as a partner] you seem like you are just supporting other than being a main player (consortium A, partner institution, M2).

Participants using the decentralised system reported that delegating managerial responsibilities empowered partners and facilitated capacity strengthening through local-level decision-making and tailored consortia processes and plans. This practice promoted partner ownership of consortium goals and helped sustain built capacity.

It helps when you give institutions some sort of power… you are building a system that will last other than having everything run from the lead institution… I think that also brings in some bit of ownership (consortium B, lead institution, M3).

We’re building their capacity to move on beyond the current consortium grant… There are partners who have actually come back to us and said, “We are getting less money from the consortium, but we’ve actually learned a lot which has enabled us to go on to bigger grants” (consortium B, lead institution, M2).

Beyond the tangible structures, many intangible factors determined the balance of power in consortia. For example, positive practices such as negotiation and consensus building, which fostered greater ownership of consortia decisions, were seen as indicators of shared power.

We see it as a participatory approach to governance as opposed to a talk-down directive. There is a kind of negotiation-based governance, in that at any given time nobody is completely so wrong to be rubbished out (consortium B, partner institution, M6).

Fully representative structures did not always result in full participation by partners. One consortium participant felt that individual agency was significant in decision-making.

I think that when it comes to influences on the board…, there is no real inequity issue there apart from that related to the individual… some of the representatives are very powerful, and that’s not a system question; it’s an individual person question (cconsortium A, partner institution, R9).

Several factors affected partners’ agency in decision-making and engagement in consortia. Partners with less research capacity and experience, who joined the consortium in the latter stages, or whose first language was not English felt inhibited and inadequate, thus hindering their full participation in decision-making. The situation becomes more complicated when a partner is faced with multiple limitations as the compounded effect makes it even more difficult to overcome the constraints.

Some people are more influential because first, they have more experience in certain areas… So, for me, I totally understand that their voice is louder than mine (consortium C, partner institution, R3).

So, your individual motivation, your passion for what we are doing, your language barriers, your institutional capacity and strengths… all these things affect your full participation (consortium C, partner institution, R4).

It emerged that the intangible aspects of consortium management were often more critical than the tangible as such factors disincentivised some partners and minimised their participation even in inclusive structures.

It’s one thing to agree on how to move forward. When the reality comes, and when the rubber hits the road as they say, then people start developing all sorts of feelings… So, the challenge is not so much the structure of governance but the real issues and the functionality (consortium B, lead institution, R2).

#### Interaction between tensions

Across the consortia, consortia experiences indicated that multiple tensions emerged during each management process (table 3). For example, when determining consortium goals, leaders have to decide between individual partner interest or collective interests (tension 1) as well as between choosing goals that are easier to deliver or goals that meet the greatest partner needs (tension 2). Leaders, therefore, had to be mindful of both the existence of and interaction between multiple tensions during decision-making.

**Table 3:** Tensions associated with different consortium management processes

Consortium management process	Tensions
Selecting partners	Based on ability to perform or capacity needs (**T2**) Based on existing capacity or which partners require capacity (**T3**)
Determining consortium goals	Emphasis on partner interests or collective interests (**T1**) Choosing goals that are easier to deliver or those that meet partners’ greatest goals (**T2**)
Instituting governance structures and processes	Emphasis on efficient decision-making or partner’s capacity that will be strengthened from participation in governance (**T2**) Power adequately shared among partners or greater control by some partners (**T4**)
Assigning roles	Based on partner’s ability to deliver or capacity that will be developed when executing role (**T2**) Based on partner’s existing capacity or partner inclusion irrespective of capacity (**T3**)
Managing partners	Centralised or decentralised systems based on quicker delivery of outputs or capacity gained by partners as they self-manage (**T2**) Shared power through decentralisation or greater control by lead partners through centralised systems (**T4**)
Allocating resources	Based on partner’s ability to deliver outputs or capacity needs (**T2**) Based on partner’s existing capacity to use resources or equitable allocation (**T3**)

**T1**—individual versus collective interests; **T2**—efficient programme delivery versus effective capacity strengthening; **T3**—excellence versus equity; **T4**—shared power versus greater control.

## Discussion

This study sought to critically examine consortium management practices among LMIC-led consortia and their effect on HRCS efforts. Findings indicate that management strategies adopted by consortia have a direct consequence on the capacity gains of partners. For example, a decentralised partner management system strengthened the capacity of partners to source for and manage their own research grants. Thus, in consortia capacity strengthening, management processes should be prioritised in similar ways as other factors such as financial and human resources. The findings also suggest that enhanced consortium management, including management of tensions, should in itself be seen as a capacity goal and outcome. Thus, it is essential to identify and unpack the capacity development implications of management tensions and the strategy options they present.

While these findings are grounded in data derived from the three consortia, the emerging concepts and framework are not discipline-specific and potentially applicable to RCS consortia beyond the health field. As such, the findings are discussed below in a broader RCS consortia context.

### Unpacking and managing tensions in consortia

Tensions reveal the underlying drivers of consortia challenges and indicate misalignments between multiple perspectives and interests.^42^ Tensions in decision-making have been discussed in the broader organisational management literature,^43–45^ and specifically regarding networks.^46–48^ Tensions between self and collective interests,^43 49^ centralised and decentralised management models,^46 50^ short-term and long-term interests^51 52^ and efficiency and effectiveness^46^ are inherent in different types of collaborations. However, the uniqueness of tension management in consortia is not in the existence or types of tensions but in how they are resolved in the context of RCS programmes. Capacity development should be the foremost deciding factor when addressing tensions in HRCS consortia. However, the complex nature of research capacity coupled with a lack of conceptual consistency in the literature have rendered RCS open to wide interpretation.^10^ There are differences in the perception and prioritisation of RCS among and within consortia—how research capacity is interpreted and strengthened and how different capacities are valued. In our study, these differences were not only drivers of tensions but also influenced the strategies that were adopted in consortia, such as overemphasising researcher training due to a perception that capacity strengthening means training. Moreover, the dynamics of tensions, their management and their effect on RCS were not always consciously or explicitly recognised. Consortia leaders did not always explicitly lay out all the options when tensions were encountered or factors driving their decisions. Consortia also did not follow a prescribed framework or set of strategies for managing tensions but solved challenges on an ad hoc basis and as they saw best, drawing on their knowledge, experience and discussions. Although they often chose to maintain a balance between conflicting options, leaders sometimes made clear decisions for one option over the other.

In the broader organisational and management literature, three common approaches to addressing tensions have been proposed: win-win, trade-off and paradox approaches.^53–55^ The win-win approach avoids the tension by focusing on areas of alignment between the competing elements, the trade-off approach eliminates the tension by weighing the pros and cons of the competing elements and making a choice and the paradox or integrative approach accepts the tensions by embracing the contradictory demands and making continuous efforts to resolve them.^54 56^ These different approaches were used at different times by the consortia studied, although implicitly. For instance, focusing on a common goal for all partners was one consortium’s way of avoiding the multiple interests and needs of partner consortia. Also, choosing a centralised partner management approach with the associated missed capacity development opportunities is a trade-off. Selecting both ‘stronger’ and ‘weaker’ partners to enable both programme performance and capacity strengthening was consortia’s demonstration of the paradox approach. There appears to be a convergence in the literature towards an agreement that, although the win-win and trade-off approaches have been dominant in practice over the years, the paradox approach appears to be better suited to sustainability aims.^54 55 57^ The first two approaches have been reported to only work in the short term, and tensions resurface over time, whereas the paradox approach pushes actors to continuously explore the tensions and devise creative and sustainable solutions.^54 56^

Tensions are nuanced and must be interpreted within the specific contexts within which they occur.^43 44^ Our findings also highlight the interconnection between tensions. The nuanced and inter-related nature of tensions and the need to continuously devise solutions means that tension management can ‘neither be formulaic nor reductionist’.^43^ Thus, proactive and explicit identification of tensions and available capacity development-oriented guidance for resolving them will serve RCS efforts better.

Our findings support the proposition that funders can play an important role by clarifying expectations regarding equity, excellence, capacity strengthening and impact.^19 58^ Such funder-supported guidance is particularly essential because consortia decisions are significantly influenced by the quest for consortia performance, which is in turn greatly influenced by funder expectations, evaluation indicators and how capacity strengthening outputs are measured. For instance, outputs such as number of persons trained were recognised as valuable deliverables, whereas efforts to enhance institutional systems were considered challenging and an encumbrance to the delivery of preferred outputs. Thus, conceptual clarity of research capacity and consortium performance in the RCS context are essential for tension management in HRCS consortia.

### RCS consortium management: are adopted strategies fit for purpose?

A theory-based assessment of strategies adopted by study consortia demonstrates the extent to which these strategies enable or hinder capacity goals. As indicated earlier, capacity is a systemic phenomenon, and its development is a complex, holistic and long-term process requiring interactions between individual, organisational and environmental levels as well as engagement of multiple dimensions such as skills, leadership, infrastructure and management systems to be effective and sustainable.^32 33 35 59^ Due to consortia’s capacity strengthening aims, management decisions should ideally be made in the light of these capacity development principles. For example, when tensions between individual and collective interests are encountered, strategies that emphasise each partner’s needs and a broader range of research capacity components are likely to serve capacity development aims more effectively. This is because partners vary, and sustainable research capacity is context specific and reliant on interactions between multiple capacity dimensions within local systems. To illustrate, prioritising some capacity dimensions, such as only training researchers (due to their tangibility and quick output delivery), will produce partial capacities if not embedded in broader and more holistic institutional capacity strengthening plans. This reductionist approach takes little account of the complexity of RCS and the fact that it is not just an aggregation of different components or simple input–output processes. Similarly, prioritising some management strategies for the sake of short-term results, such as centralised management systems or creation of parallel grant management processes instead of institutionalising these processes locally, undermine capacity strengthening within partner contexts. Although the latter approach may appear less efficient with risks to programme reporting and performance, it enables self-organisation and drives effective and sustainable capacity development.

Furthermore, the hinging of consortia decisions solely or heavily on excellence to the detriment of equity connotes a result-based ideology more than the need-based and relevance-driven thinking that undergirds research capacity development.^60^ Indeed, some funders have acknowledged that ‘excellence’ and how it is measured needs to be reconceptualised, and excellence and equity considered as linked rather than competing elements in RCS decision-making.^58 61^ However, unless such calls are backed by explicit statements in funder policies and strategies, and subsequently operationalised when reviewing funding applications and consortium reports, the status quo is likely to remain. Consortia leaders will be inclined towards maximising programme performance based on easy-to-measure evaluation indicators, and capacity strengthening will continue to be undermined.

Another prevalent issue in consortia is the distribution of power across partners.^62–65^ At the heart of capacity, development is empowerment.^66^ Thus, capacity strengthening will thrive in consortia with balanced power relations demonstrated by shared finances, expertise, leadership and access to resources and networks^67^; and where partners possess the power to self-organise and adapt RCS activities to their own contexts.

Overall, it is evident that purposeful consortium management, including management of tensions, is a capacity strengthening goal in itself. The ability to understand the role of management processes and strategies is a key component of research capacity. In addition, management of HRCS consortia needs to differ from management of organisations or even purely research consortia due to their primary capacity development mandate. When management strategies align with capacity development tenets, consortium management processes become capacity strengthening mechanisms for participating individuals and institutions. Adopting strategies that are fit for the RCS purpose ensures programme effectiveness and value for money,^68^ and funders are best placed to facilitate this through appropriate guidelines.^19^

### The tangible is powered by the intangible

Capacity constitutes both tangible and intangible dimensions such as infrastructure and culture, respectively.^16 69^ The study findings demonstrate that the intangible aspects of consortium management such as power relations, actor agency and ownership are at least as critical as the tangible structures put in place. However, the significance of the tangible–intangible interaction and its influence on capacity outcomes have not fully suffused management practice.^18^ For example, consortia commonly use representative governance structures to ensure inclusion and power sharing without addressing the intangible barriers such as feelings of inadequacy and lack of ownership, which disincentivise and disempower some partners and undermine full inclusion and power balance. The importance of this interdependency has been recognised in business partnerships where emphasis is placed on going beyond formal governance structures to fostering collaborative relationships and behaviour to attain the desired goals.^70 71^ Our findings suggest that tangible management structures and processes need to be facilitated by intangible managerial ‘software’ such as communication, inclusion, openness and commitment to learning to ensure that the intent of adopted strategies is realised. These intangible elements need to be explicitly identified and purposefully promoted to enhance the capacity strengthening role of consortia processes.

### Framework for managing HRCS consortia

**Figure 2:**
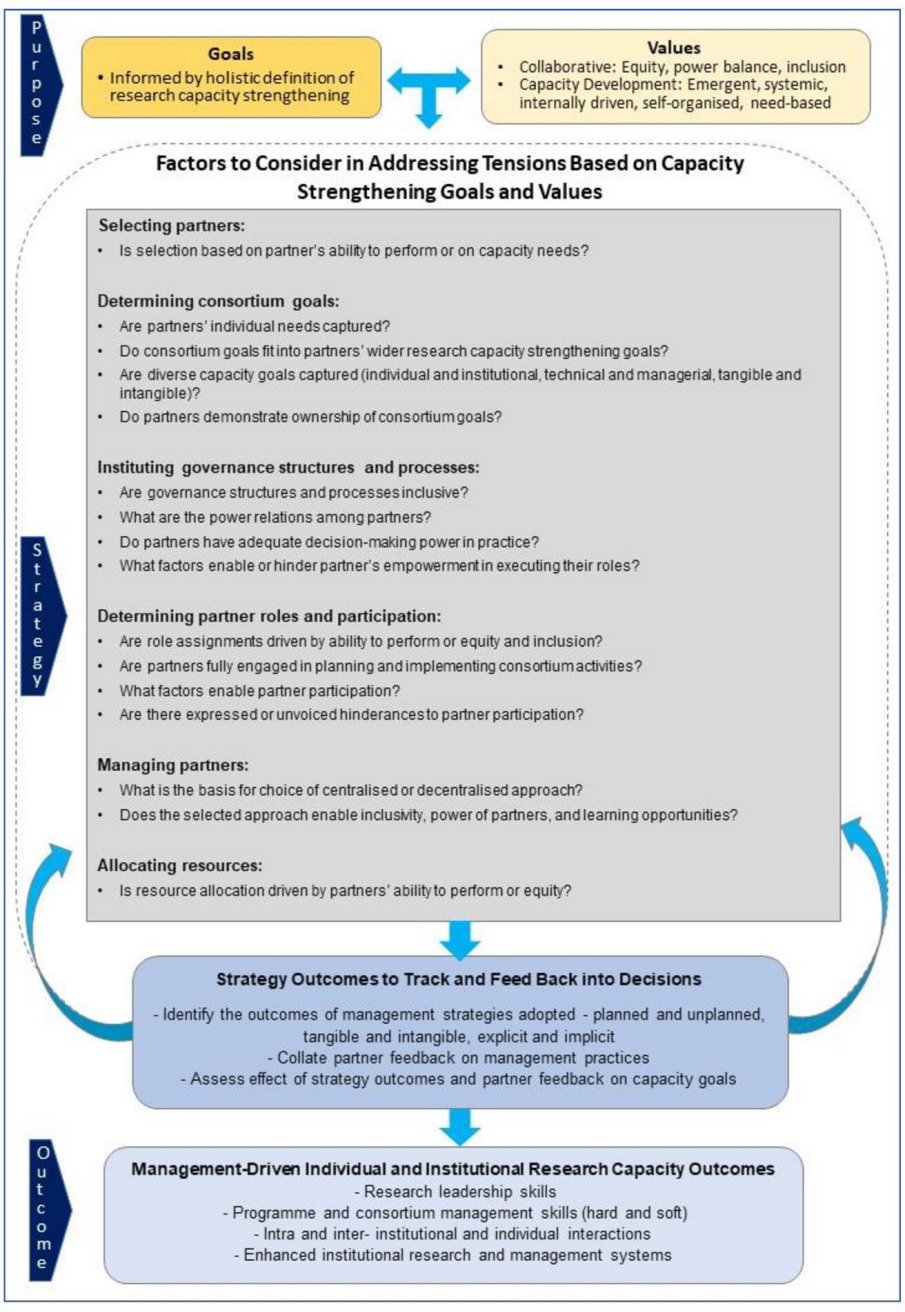
Steps and factors that should be considered in consortium management to promote capacity strengthening.

We draw on the study findings to propose a framework to support decision-making in consortia with capacity strengthening aims (figure 2). This framework takes cognizance of: (1) the capacity strengthening purpose of consortia, (2) the multilevel and multidimensional nature of capacity, (3) the tangible and intangible aspects of consortium management, (4) ‘how’ management processes are executed and not just ‘what’ processes are followed and (5) the capacity strengthening value of consortium management processes and practices. The framework represents a ‘theory of change’^72^ and maps out a fresh approach to consortium management based on a conceptual understanding of capacity development and aimed at optimising research capacity gains derived from management processes. The questions in the framework are derived from the identified tensions from the study and categorised under the different consortium management processes.

The framework is intended to be a guidance tool for consortia as they make management decisions. Leaders could consider the questions in the framework to ensure that they are mindful of the driving factors behind their choices, and ideally, capacity development is prioritised during management processes. For example, when selecting partners, leaders can reflect on whether the selection is based on ability to perform or capacity needs of potential partners. The decisions are, thus, made with a full awareness of the capacity development implications of choices made. The framework first establishes consortia’s capacity strengthening goals as well as the collaborative and capacity development values that undergird RCS consortia and need to guide decision-making. Consortia’s management strategy options can then be vetted for alignment with these established goals and values. For instance, consortia goals should be need based and aim for equitable capacity benefits across partners. Both tangible and intangible managerial elements need to be considered to ensure that strategies enable capacity development in partners. Furthermore, consortia need to constantly assess emerging tensions and any contextual influences, actively track the effect of adopted strategies on learning goals and feed these back into decision-making. These will ensure that capacity opportunities are not missed, any hindrances are timeously addressed and the desired management-driven capacity outcomes are maximised.

Questions raised in the framework are not meant to promote dichotomous strategy choices but to draw attention to critical considerations and the capacity implications of strategy options during decision-making.

We acknowledge that implementing the framework would not be without challenges considering that the factors which have precipitated its need, such as funders’ influence and the overemphasis on quantifiable evaluation indicators, may still dominate decision-making. The framework is intended to consistently draw attention to the centrality of capacity development in managerial deliberations and decisions. Additionally, the framework will require validation through empirical testing to refine and enhance its applicability. As an initial step, we presented the framework to senior consortia stakeholders and funders to elicit their perception and ascertain its relevance and applicability to their experiences. The positive feedback on its practical value received from these potential users is an indicator of its relevance for consortia.

### Recommendations for policy and practice

To attain greater and more sustainable capacity gains from RCS initiatives, it will be important to continuously reorient RCS policy and practice to reflect emerging evidence. Box 1 outlines evidence-informed recommendations to consider in designing and implementing RCS programmes. It is important to recognise the reality and capacity implications of tensions in consortium management.

Box 1 **Recommendations for research capacity strengthening (RCS) programmes**

1. Acknowledge the reality and capacity implications of tensions in consortium management and how compromises and trade-offs undermine capacity strengthening.
2. Base RCS programme requirements and management decisions on a holistic perception of research capacity to maximise capacity strengthening.
3. Clarify the primacy of the RCS aims of the programme and ensure that it permeates through programme design and reporting requirements.
4. Apply RCS-specific outputs, outcomes and indicators to programme evaluation processes to promote prioritisation of capacity-strengthening principles.
5. Recognise that consortium management processes are capacity-strengthening mechanisms that merit adequate resources and time.
6. Accept that capacity development is ‘risky’ as it is systemic, requires time and cannot always be measured or quantified in the short term.
7. Back commitments to capacity strengthening with clear policies and guidelines to empower consortia and give them the latitude to choose appropriate management strategies.
8. Promote the generation and use of empirical evidence in RCS practice to improve programme design, implementation and outcomes

The pivotal role of the interpretation of RCS demands a consensus among RCS policymakers, funders and implementers on a more holistic perception of research capacity, which should then reflect in the design of programmes. The capacity-strengthening aim of RCS consortia should be visibly prioritised by funders and consortia alike, so that it becomes the fulcrum around which management decisions revolve. Furthermore, the role of evaluation indicators in tension management, particularly in spurring trade-offs, demands a redefinition of performance for RCS consortia. What is mandated must match what is measured. Programmes and their evaluations must cover a wide range of capacity changes, including quantifiable and unquantifiable, tangible and intangible, technical and managerial and whether wholly or partially attributable to the programme. Using such a wide lens will ensure that all types of capacities required for research, including managerial capacity, are identified, planned for, resourced, tracked and evaluated, so that some capacity opportunities and gains are not overlooked. In addition, consortia leaders should endeavour to provide comprehensive feedback to funders, even when not stipulated in reporting requirements. For example, highlighting management tensions, the resulting trade-offs and how decisions enable or hinder capacity strengthening will increase stakeholder awareness of implementation realities and outcomes. Consortia feedback will then serve as a source of learning for funders and programme initiators. While research in this area is emerging, it is still underdeveloped and needs greater attention. Thus, combining such learning with a well-supported research component will significantly enhance the effectiveness and sustainability of the RCS agenda.

### Study strengths and limitations

This study aimed to provide empirical evidence to inform HRCS practice.^10 18^ The study drew on a capacity development lens to examine consortium management practices, ensuring that the proposed framework has both theoretical and empirical bases. Additionally, we employed multiple strategies to ensure research rigour and trustworthiness, including using the multiple case study design,^73 74^ data and method triangulation^75^ and peer debriefing.^76^ We acknowledge that the study’s focus on consortia in one Africa-based HRCS initiative and the potential influence of social desirability biases on the research process^77^ present some limitations. It is worth noting that the goal of this study was not to attain generalisability of the findings to all consortia, but rather to enhance the potential transferability of the findings and analytical generalisability of the emerging ideas and concepts to similar contexts.^78 79^ To further strengthen the evidence in the field, it would be valuable to examine the management processes and practices of consortia led by institutions from high-income countries and those in different geographical settings to capture other contextual influences. In addition, it will be necessary to validate and build on the proposed framework (figure 2) through empirical testing with other RCS consortia. Finally, this study has highlighted several potential areas of research on RCS more broadly, including the need for RCS-specific definitions of excellence and performance and a broad range of evaluation outcomes and indicators for assessing RCS initiatives.

## Conclusion

It is evident that a critical aspect of consortium management is the identification and handling of tensions between very compelling strategy options. Thus, decision-making in consortia requires a constant navigation of these tensions and strategy choices that have capacity development consequences. There is no ‘one size fits all’ formula for managing consortia as contexts vary. However, we have proposed an evidence-informed framework, which highlights potential tensions and provides RCS-specific guidance on where priorities should be placed to ensure that management decisions are weighted towards the overarching RCS goals. Consortium management processes and practices are inextricably linked with consortia outcomes. Indeed, at a time when attention on the need to strengthen equity in global health capacity is heightened, having blind spots to the cruciality of management capacity in HRCS poses the risk of entrenching current inequities rather than transforming them. Hence, leveraging the capacity strengthening opportunities in management processes will maximise the returns on investments made and contribute to broader global health goals.

## Data Availability

Data may be obtained from a third party and are not publicly available. Access to underlying data is restricted to the study team for ethical reasons. This restriction was highlighted during the ethical approval process due to the small number and unique characteristics of participating consortia and the potentially sensitive nature of the information collected. Anonymisation of the raw data does not adequately ensure the protection of study participants and thus will not be shared beyond the team. Specific requests can be submitted to the KWTRP Data Governance Committee via email: dgc@kemri-wellcome.org.
