# Supplementary material for: A framework for managing health research capacity strengthening consortia: addressing tensions and enhancing capacity outcomes
Source: BMJ Glob Health. 2022 Oct 3;7(10):e009472. doi: 10.1136/bmjgh-2022-009472 (PMC9535163; doi:10.1136/bmjgh-2022-009472)
Supplement: Supplementary data [file bmjgh-2022-009472supp001.pdf]

CONSORTIUM MANAGEMENT CASE STUDY

SEMI-STRUCTURED IN-DEPTH INTERVIEW GUIDES

A. Consortia Directors

Understanding of HRCS

1. How would you personally interpret the term/concept 'research capacity strengthening'? What comes to mind?
2. What are your institutional research capacity strengthening priorities?
   - Any tensions between them, e.g., does developing one type take away from the other?
3. To what extent does this thinking align with the DELTAS approach?
   - Activities? Expected outputs? Practices?
   - Are there other areas of capacity that require attention?

Influence of HRCS thinking on key management processes

4. How have adopted management strategies worked?

   i) Governance structures and processes (Boards, committees, secretariat)
   ii) Financial management
   iii) Resource allocation/trainee award strategy
   iv) Reporting and evaluation

5. Why did the consortium choose a centralised/decentralised approach?
6. Are there any differences in the way the consortium is managed because of the focus on HRCS?

   i) Selecting partners
   ii) Deciding on consortium goals
   iii) Determining partner roles and responsibilities
   iv) Establishing governance structures and practices
   v) Allocating resources (funds) including trainee awards
   vi) Financial management structures and processes
   vii) Programmatic management structures and processes

   Any dilemmas encountered?

7. Are there any differences in the way the consortium is managed because of this specific initiative or funder or compared to any others you've managed? How? Why?

Effect of management processes on capacity development

8. What is your perception of the theory of change/LFA approach to measuring/evaluating capacity strengthening? How can it be improved?

9. Are there other ways that capacity strengthening happens that are not recognised? How are you able to identify that capacity strengthening is happening?

10. Do consortium management processes play any role in the capacity strengthening process?

11. What is the effect of consortium management processes on individual and institutional capacity?
    - Which aspects enable and which ones hinder?
    - Examples?

12. To what extent are consortium management processes embedded in the institution?
    - Partner selection, goal development, governance, programme and financial management

Recommendations for HRCS models and management approaches

13. Based on your thinking and experience, what kind of HRCS programme design will you propose for an initiative like this if given the opportunity?

14. What kind of management approach will you propose that supports capacity development?
    - Partner selection and management
    - Programmatic
    - Financial
    - Reporting
    - Evaluation

B. Managers at Lead Institutions (Programme, M&E, Finance)

Programmatic management processes

1. When did you join the consortium, and what are your responsibilities?
2. What processes are used to manage the programmatic aspects of the consortium, i.e. activity planning, coordination and monitoring
   - What influenced the choice of these processes?
   - What benefits and challenges have you encountered in using these processes?

Understanding of HRCS

3. How would you personally interpret the term/concept 'research capacity strengthening'? What comes to mind?
4. Which areas should your institution prioritise in strengthening its research capacity?
5. What do you think are the best ways by which these capacity areas can be developed?
   - Approaches? Activities? Practices? Types of resources?
6. How does your personal and/or institutional thinking align with the DELTAS approach?
   - Activities? Expected outputs? Practices?
   - Are there other areas of capacity that require attention?
7. What are some of the dilemmas that you have encountered due to your institutional thinking on HRCS and that of DELTAS, if divergent?

Influence of HRCS thinking on key management processes

8. How have adopted management strategies worked?
   i) Governance structures and processes (Boards, committees, secretariat)
   ii) Financial management
   iii) Resource allocation/trainee award strategy
   iv) Reporting and evaluation
9. Are there any differences in the way the consortium is managed because of the focus on HRCS?
   i) Selecting partners
   ii) Deciding on consortium goals
   iii) Determining partner roles and responsibilities
   iv) Establishing governance structures and practices
   v) Allocating resources (funds) including trainee awards
   vi) Financial management structures and processes
   vii) Programmatic management structures and processes

   Any dilemmas encountered?
10. Are there any differences in the way the consortium is managed because of this specific initiative or funder or compared to any others you've managed? How? Why?

Effect of management processes on capacity development

11. What is your perception of the theory of change/LFA approach? Is it adequate, and how can it be improved?

12. Are there other ways that capacity strengthening happens that are not recognised? How are you able to identify that capacity strengthening is happening?

13. Do consortium management processes play any role in the capacity strengthening process?

14. What role have the consortium management processes played in individual and institutional capacity development?
    - Which aspects enable and which ones hinder?
    - Examples?

15. To what extent are consortium management processes embedded in the institution?
    - Partner selection, goal development, governance, programme and financial management

Recommendations for HRCS models and management approaches

16. Based on your thinking and experience, what kind of programme design will you propose for an HRCS initiative if given the opportunity?
    - Model, implementer, goals, activities?

17. What management approach will you propose?
    - Partner management
    - Programmatic
    - Financial
    - Reporting
    - Evaluation

C. Leaders at Partner Institutions

Partner perception of consortium management processes

1. What are your responsibilities?
2. What motivated your institutions' participation in this consortium?
3. How were the consortium goals determined and agreed upon?
   - How are your institutional goals represented?
   - Are there others that you would have wanted to be represented?
4. I understand the consortium has/uses X (management structure and processes) …
   - How has it functioned? How can they be improved? Can anything be done differently?
5. I understand consortium resources are allocated based on X (including trainee award strategy), how has this worked so far?
6. I learnt from my first phase of interviews that … (financial management approach) … what are the advantages and disadvantages of this approach?
7. What are your thoughts on partner relationships in the consortium?
   - How are equity, power balance, and inclusivity promoted?
8. What is your general perception of how the consortium is managed?
9. What influences are exerted by the lead/partners on consortium processes and interactions?
10. Are there any differences in the way the consortium is managed compared to others you've participated in?

Understanding of HRCS

11. How would you personally interpret the term/concept 'research capacity strengthening'? What comes to mind?
12. What are your institutional research capacity strengthening priorities?
13. How does this thinking align with the DELTAS approach/the consortium? Are there any divergences?
14. Were these needs and approaches brought up during DELTAS discussions, and how were they incorporated into the consortium's goals?
15. Are there other HRCS approaches or activities you would have proposed?

Effect of management processes on capacity development

16. Are there any differences in the way the consortium is managed because of the focus on RCS?
17. What is your perception of the theory of change/LFA approach? Is it adequate, and how can it be improved?

18. Are there other ways that capacity strengthening happens that are not recognised or reported?
19. Do consortium management processes play any role in the capacity strengthening process (i.e. contribute or a toll on)? How does the way the consortium is managed affect capacity strengthening?
20. What is the effect of consortium management processes on:
    - Individual capacity
    - Institutional capacity
21. To what extent are consortium management processes embedded in the institution?
    - Partner selection, goal development, governance, programme and financial management

Recommendations for HRCS models and management approaches

22. Based on your thinking and experience, what kind of programme design will you propose for an HRCS initiative if given the opportunity?
    - Model, goals, activities?
23. What will be the best way of approaching the following management processes for an HRCS consortium and why?

    viii) Selecting partners
    ix) Deciding on consortium goals
    x) Determining partner roles and responsibilities
    xi) Establishing governance structures and practices
    xii) Allocating resources (funds) including trainee awards
    xiii) Financial management structures and processes?
    xiv) Programmatic management structures and processes?
    xv) Reporting and evaluation

D. Managers at Partner Institutions (Programme, M&E, Finance)

Partner perception of consortium management processes

1. What does your role entail?
2. I understand the consortium has/uses X (management structure and processes, including programmatic management) …
   - How has it functioned?
   - What is your perception (has been your experience) with the management processes adopted?
   - How can they be improved?
3. I understand consortium resources are allocated based on X (including trainee award strategy), how has this worked so far?
4. I learnt from my first phase of interviews that … (financial management approach) … what are the strengths and challenges of this approach?
5. What are your thoughts on partner relationships in the consortium?
   - How are equity, power balance, and inclusivity promoted?
6. What is your general perception of how the consortium is managed?

Understanding of HRCS

7. How would you personally interpret the term/concept 'research capacity strengthening'? What comes to mind?
8. Which areas should your institution prioritise in strengthening its research capacity?
9. What would you propose as the best ways of developing these capacities?
   - Approaches? Activities? Practices? Types of resources?
10. How does this thinking compare to the DELTAS approach? Are there any divergences?
    - Activities? Expected outputs? Practices?
11. Are these needs and approaches brought up during consortium discussions, and how are they incorporated into the consortium's activities?

Effect of management processes on capacity development

12. Are there any differences in the way the consortium is managed because of the focus on HRCS?
13. What is your perception of the theory of change/LFA approach? Is it adequate, and how can it be improved?
14. Are there other ways that capacity strengthening happens that are not recognised? How are you able to identify that capacity strengthening is happening?
15. Do consortium management processes play any role in the capacity strengthening process?

16. What role have the consortium management processes played in the development of individual and institutional capacity?
    - Which aspects enable and which ones hinder?
    - Examples?

17. To what extent are consortium management processes embedded in the institution?
    - Partner selection, goal development, governance, programme and financial management

Recommendations for HRCS models and management approaches

18. Based on your thinking and experience, what kind of HRCS programme design will you propose if given the opportunity?
    - Model, implementer, goals, activities?

19. What will be the best way of approaching the following management processes and why?
    i) Selecting partners
    ii) Deciding on consortium goals
    iii) Determining partner roles and responsibilities
    iv) Establishing governance structures and practices
    v) Allocating resources (funds) including trainee awards
    vi) Financial management structures and processes?
    vii) Programmatic management structures and processes?
    viii) Reporting and evaluation

E. Other Institutional Stakeholders at Lead and Partner Institutions

Participation and goals

1. What is your role in the consortium?

2. What motivated your institution's participation in this consortium?

3. To what extent are your institutional goals represented in the overall consortium goals?
   - Are there others that you would have wanted to be more represented?

4. What is your perception of partner relationships in the consortium?
   - How are equity, power balance, and inclusivity promoted?

5. Have there been any critical incidents (e.g. unintended occurrences or changes, e.g. personnel), and what was the effect and response?

Understanding of HRCS

6. How would you personally interpret the term/concept 'research capacity strengthening'? What comes to mind?

7. Which areas does your institution need to prioritise in its bid to strengthen its research capacity?

8. How does your personal and/or institutional thinking compare to the DELTAS approach?
   - Has it been explicitly discussed? Are there any divergences?
   - Are there other areas of capacity that require attention or being addressed but not recognised?

9. How does this thinking compare to the DELTAS approach?
   - Activities? Expected outputs? Practices?
   - Are there other areas of capacity that require attention, or being developed but not recognised/rewarded?

Influence of HRCS thinking on key management processes

10. Are there any differences in the way the consortium is managed because of the focus on HRCS?
    i) Selecting partners
    ii) Deciding on consortium goals
    iii) Determining partner roles and responsibilities
    iv) Establishing governance structures and practices
    v) Allocating resources (funds) including trainee awards
    vi) Financial management structures and processes?
    vii) Programmatic management structures and processes?

    Any dilemmas encountered?

Effect of management processes on capacity development

11. What is your general perception of consortium management processes?
12. What role have consortium management processes played in the development of individual and institutional capacity?
    - Which aspects enable and which ones hinder?
    - Examples?
13. What is your perception of the theory of change/LFA approach? Is it adequate, and how can it be improved?
14. Are there other ways that capacity strengthening happens that are not recognised? How are you able to identify that capacity strengthening is happening?
15. To what extent are consortium management processes embedded in the institution?
    - Partner selection, goal development, governance, programme and financial management

Recommendations for HRCS models and management approaches

16. Based on your thinking and experience, what kind of programme design will you propose for an HRCS initiative if given the opportunity?
    - Implementer, goals, activities?
17. What management approach will you propose?
    - Programmatic, financial, reporting, evaluation?

F. Leaders at High-Income Partner Institutions

Partner perception of consortium management processes

1. What is your role in the consortium, and what does it entail?
2. What motivated your participation in this consortium?
3. How were the consortium goals determined and agreed upon?
   - How are your institutional goals represented?
   - Are there others that you would have wanted to be represented?
4. What is your general perception of how the consortium is managed?
5. I understand the consortium has/uses X (management structure and processes) …
   - How has it functioned so far?
   - What is your perception (has been your experience) with the management processes adopted?
   - How can they be improved?
6. I understand consortium resources are allocated based on X (including trainee award strategy), how has this worked so far?
7. What is your perception of partner relationships in the consortium?
   - How are equity, power balance, and inclusivity promoted?
8. Have there been any critical incidents (e.g. unintended occurrences or changes, e.g. personnel), and what was the effect and response?

Understanding of HRCS

9. How would you personally interpret the term 'research capacity strengthening'? What comes to mind?
10. How does this thinking compare to the DELTAS approach? Are there any divergences?
    - Activities? Expected outputs (any intangible or unrecognised)? Practices?
11. Does participation in this consortium strengthen your institution's research capacity? How?
12. Were these needs brought up during DELTAS discussions, and how were they incorporated into the consortium's goals?
13. Did/do you encounter any dilemmas as this programme was being developed or implemented?

Effect of management processes on capacity development

14. What is your perception of the theory of change/LFA approach to measuring/evaluating capacity strengthening? How can it be improved?
15. Are there other ways that capacity strengthening happens that are not recognised? How are you able to identify that capacity strengthening is happening?
16. Do consortium management processes play any role in the capacity strengthening process (i.e. contribute or a toll on)?

17. What is the effect of consortium management processes on individual and institutional capacity?
    - Which aspects enable and which ones hinder?
    - Examples?

18. To what extent are consortium management processes embedded in your institution?
    - Partner selection, goal development, governance, programme and financial management

Recommendations for HRCS models and management approaches

19. Based on your thinking and experience, what kind of HRCS programme design will you propose if given the opportunity?
    - Implementer, goals, activities?

20. What will be the best way of approaching the following management processes and why?

    i) Selecting partners
    ii) Deciding on consortium goals
    iii) Determining partner roles and responsibilities
    iv) Establishing governance structures and practices
    v) Allocating resources (funds) including trainee awards
    vi) Financial management structures and processes?
    vii) Programmatic management structures and processes?
    viii) Reporting and evaluation
