# Supplementary material for: A framework for managing health research capacity strengthening consortia: addressing tensions and enhancing capacity outcomes
Source: BMJ Glob Health. 2022 Oct 3;7(10):e009472. doi: 10.1136/bmjgh-2022-009472 (PMC9535163; doi:10.1136/bmjgh-2022-009472)
Supplement: Supplementary data [file bmjgh-2022-009472supp002.pdf]

Author Reflexivity Statement

AREA	QUESTION	RESPONSE
Study conceptualisation	1. How does this study address local research and policy priorities?	This study was nested in the Africa-led DELTAS Initiative aimed at strengthening research capacity in Africa. The study aims to strengthen the management of LMIC-led consortia and enhance the role of these consortia in strengthening local research capacity.
	2. How were local researchers involved in study design?	The first author was the Principal Investigator of the study, which was conducted as part of her PhD. The first author is from and based in an LMIC. The first author led the conceptualisation and design of the study and worked with the other study team members who acted as mentors. One co-author is from an LMIC, another has been based in an LMIC for over 25 years.
Research management	3. How has funding been used to support the local research team(s)?	The funding for this study was solely geared towards strengthening the first author's research capacity through the implementation of this study and other training and skills building activities
Data acquisition and analysis	4. How are research staff who conducted data collection acknowledged?	All the data was collected by the first author
	5. How have members of the research partnership been provided with access to study data?	The data is owned by the LMIC institution which hosted the first author. The data is accessible to every member of the study team and processes for accessing the data is coordinated by the LMIC authors
	6. How were data used to develop analytical skills within the partnership?	The building of analytical skills of the first author was an important aim of this research process. The study team held regular data analysis meetings and the team analysis approach enhanced the analytical skills of not just the first author but all members of the team.
Data interpretation	7. How have research partners collaborated in interpreting study data?	The study team held regular data analysis meetings to analyse the data and agree on the findings. The first author presented the data and any emerging themes and all the authors discussed and interpreted the results.

Drafting and revising for intellectual content	8. How were research partners supported to develop writing skills?	The first author developed the first draft of the manuscript and all authors reviewed and edited until a final draft was approved by the whole team.
Authorship	9. How will research products be shared to address local needs?	The study findings have been presented to the participating consortia, funders and other research capacity strengthening stakeholders. A report on the findings have also been shared with participating consortia. A policy brief, recommending ways to ensure that consortia enhance local capacities, is being developed to share with research capacity policymakers, funders, and programme designers and implementers.
	10. How is the leadership, contribution and ownership of this work by LMIC researchers recognised within the authorship	The first author is an LMIC researcher, and all study team members are co-authors.
	11. How have early career researchers across the partnership been included within the authorship team?	The first author is the early career researcher in the team
	12. How has gender balance been addressed within the authorship?	The authors are made up of 50% men and 50% women
Training	13. How has the project contributed to training of LMIC researchers	The study was part of the PhD project of the first author and was aimed at primarily enhancing her research training. The study was nested within the Africa-led DELTAS Initiative aimed at strengthening research capacity in Africa.
Infrastructure	14. How has the project contributed to improvements in local infrastructure	This project has not directly contributed to improvements in local infrastructure
Governance	15. What safeguarding procedures were used to protect local study participants and researchers?	The study participants were primarily leaders and managers of Africa-led consortia who were given the opportunity to freely decide whether to participate in the study or not. The consent process also included ensuring that all study participants exercised their autonomy in participating in the study.
